# Composition and short-term stability of gut microbiota in lean and spontaneously overweight healthy Labrador retriever dogs

**DOI:** 10.1186/s13028-022-00628-z

**Published:** 2022-03-28

**Authors:** Josefin Söder, Sara Wernersson, Katja Höglund, Ragnvi Hagman, Sanna Lindåse, Johan Dicksved

**Affiliations:** 1 Department of Clinical Sciences, Faculty of Veterinary Medicine and Animal Science, Swedish University of Agricultural Sciences, Box 7054, 75007 Uppsala, Sweden; 2 Department of Anatomy, Physiology and Biochemistry, Faculty of Veterinary Medicine and Animal Science, Swedish University of Agricultural Sciences, Box 7011, 75007 Uppsala, Sweden; 3 Department of Animal Nutrition and Management, Faculty of Veterinary Medicine and Animal Science, Swedish University of Agricultural Sciences, Box 7024, 75007 Uppsala, Sweden

**Keywords:** 16S rRNA sequencing, Canine, Faeces sampling, Obesity, Temporal variations

## Abstract

**Background:**

The gut microbiota and its metabolic end-products act in close collaboration with the nutrient metabolism of the animal. A relationship between excess adiposity and alterations in gut microbiota composition has been identified in humans and rodents, but data are scarce for overweight dogs. This study compared composition and temporal variations of gut microbiota in healthy lean and spontaneously overweight dogs. The analysis was based on three individual fresh faeces samples from each dog during a 10-day period. Twenty-seven healthy and intact male Labrador retriever dogs were included, 12 of which were classified as lean (body condition score (BCS) 4–5 on a 9-point scale) and 15 as overweight (BCS 6–8). Gut microbiota was analysed by Illumina sequencing of the V3-V4 region of the 16S rRNA gene.

**Results:**

Lean and overweight groups of dogs were not separated by principal coordinate analysis (PCoA), analysis of similarity (one-way ANOSIM, P = 0.99) or species indicator analysis (IndVal) using operational taxonomic units (OTU) data. Gut microbial taxa at phylum, family or genus level did not differ between lean and overweight dogs in mixed-model repeated measures analyses. Short-term stability, evaluated by similarity index, did not differ between lean and overweight dogs over the 10-day period. Pooled Firmicutes/Bacteroidetes (F/B) ratio was 3.1 ± 3.7 in overweight dogs and 2.1 ± 1.2 in lean dogs (P = 0.83). Individual dogs, irrespective of body condition (lean or overweight), displayed variation in mean alpha diversity (Chao-1 index range 122–245, Shannon index range 2.6–3.6) and mean similarity index (range 44–85%).

**Conclusions:**

Healthy lean and spontaneously overweight Labrador retriever dogs had comparable gut microbiota composition and short-term stability over a 10-day sampling period. There were no alterations in microbial diversity or in relative abundance of specific taxa at phylum, family or genus level in overweight compared to lean dogs. Our findings suggest that there are few detectable differences in gut microbiota composition between healthy spontaneously overweight and lean dogs by the current method. Future application of metagenomic or metabolomic techniques could be used to investigate microbial genes or microbial end-products that may differ even when microbiota compositional analyses fail to detect a significant difference between lean and overweight dogs.

**Supplementary Information:**

The online version contains supplementary material available at 10.1186/s13028-022-00628-z.

## Background

Well-being and longevity of pet dogs are major concerns for dog owners, as dogs often are regarded as family members in today’s society [1]. Overweight and obesity in dogs play a crucial role in their well-being and longevity, as excess adiposity causes chronic diseases [2], shortens the lifespan [3–5] and decreases quality of life in dogs [6, 7]. The prevalence of canine overweight is generally considered to be about 30–40% worldwide [8–11], but there are indications of increasing prevalence [12]. A potential reason is the shared sedentary lifestyle by dog owners and their dogs [11, 13, 14] following the trend in the human obesity epidemic [15].

Nutritional management is important to maintain dogs in balanced body condition and at normal body weight, but the diet also affects gut microbiota composition [16]. A relationship between gut microbiota alterations and overweight in humans and in rodent models has been well-established [17–19], but gut microbiota composition in overweight dogs has been less investigated. The few studies published so far demonstrate considerable individual variation in the gut microbiota of pet dogs [20, 21], and indicate a lower gut microbial diversity in obese compared with lean dogs [21–24].

In obese humans, high dominance of Firmicutes in relation to Bacteroidetes has been shown [18]. As human and canine gut microbiota show similarities in composition and in response to dietary interventions [25] this shift in proportion might be relevant for dogs as well. The relative proportions of these phyla, commonly described as Firmicutes/Bacteriodetes (F/B) ratio, has been shown to be important for energy harvest from the diet [18, 19] and are therefore of interest in studies of overweight. Elevated F/B ratio is a quite well-established feature of the gut microbiota in overweight humans [18], but only a few canine studies have so far reported elevated F/B ratio in overweight compared to lean dogs [23, 24]. For instance, it was shown that six healthy Beagle dogs overfed a high-fat diet (33% fat given at 150% of total metabolisable energy, ME) displayed an initial but transient peak (2–3 times fold change) in F/B ratio after 4 weeks of feeding, together with a simultaneous increase in body condition [24]. In another study, 20 obese pet dogs underwent a weight loss programme with a high-fibre diet, and the study reported a decreased F/B-ratio (about 9 times fold change) at ideal body weight compared with the obese state [23].

Interactions between body condition and diet have been suggested to influence gut microbiota composition in dogs [25, 26]. For example, the similarity coefficient as an indication of microbiota resilience, was lower in six obese compared with six lean Beagle dogs when they were switched between two isocaloric diets of high or low protein content [26]. Moreover, a weight loss programme on a high-fibre, high-protein diet changed the gut microbiota composition in 20 obese pet dogs [23]. Some indication of short-term adaptation of the canine gut microbiota to dietary changes has also been found, such as a transient increase in colonic permeability as an early response to a high-fat diet [24]. In overweight humans, the gut microbiota may be less resilient to dietary changes than in lean subjects [27]. However, the combined effects of overweight and diet on gut microbiota composition, stability or diversity are not yet fully understood in dogs. Temporal variations in gut microbiota composition in lean and spontaneously overweight dogs have not been described in observational studies, as the most commonly used approach in research to date has been to modulate the diet and/or body weight of the canine subjects. In the present study, healthy intact adult dogs of one breed and sex, but differing in body condition, were included. The aim was to compare gut microbiota composition and temporal variations in healthy lean and spontaneously overweight Labrador retriever dogs, by repeated faeces sampling and analysis without any type of intervention.

## Methods

### Recruited Labrador retriever dogs

Privately-owned intact male Labrador retriever dogs were recruited by personal letters to dog owners, using a register provided by the Swedish Kennel Club. The selection process consisted of an on-line survey and a clinical health examination, including blood and urine analyses, as previously reported [28]. The exclusion criteria were: previous or present systemic or organ-related disease and treatment with antibiotics, non-steroid anti-inflammatory drugs, steroids, deworming drugs and/or proton pump inhibitors within 3 months of participation. A total of 27 healthy Labrador retriever dogs of different body condition were recruited for the study. In addition to the health examination, all dogs were weighed and photographed and their body condition score (BCS) was determined, by the same assessor, according to a 9-point scale and applying the recommended cut-off for overweight (BCS ≥ 6) [29]. Based on BCS, a lean group (BCS 4–5) consisting of 12 dogs and an overweight group (BCS 6–8) consisting of 15 dogs were established. Group age was 5.3 ± 1.4 years (mean ± SD) for the lean dogs and 5.3 ± 1.7 years for the overweight dogs. Body weight was 34.8 ± 2.5 kg (mean ± SD) for the lean group of dogs and 39.8 ± 4.7 kg for the overweight dogs, a statistically significant difference (P = 0.004).

### General study design

Included dogs were housed in their home environment and no changes were made to their regular daily exercise given by their respective owner. No adjustments were made to the dogs’ regular home diet or treats prior to participation in the study or during the study period. No intervention, neither for weigh gain nor weight loss, was performed. All included overweight dogs had spontaneously arisen overweight that had been constant for at least 3 months prior to the study according to the dog owners. During the 10-day faeces sampling period, dietary history was recorded in daily food diaries completed by the dog owner (see Additional file 1 for details). According to their daily food diaries, all dogs were fed twice a day with dry (n = 26) or wet (n = 1) complete commercial diets, the most common protein source was chicken and a limited number of dogs was fed a commercial low-fat calorie-restricted diet. The frequency with which lean and overweight dogs were awarded table scraps, treats or dog chews did not differ between the two body condition groups during the 10-day faeces sampling period (Additional file 1). The dog owners were asked to collect spontaneous fresh faeces samples from their dogs, immediately after drop on the ground, on three occasions over the 10-day sampling period (days 1, 5 and 10). These samples were placed in stool collection tubes and then frozen at − 20 °C in the home environment for a maximum of 10 days. As the dogs arrived to the veterinary clinic for clinical health examination, the faeces samples from all dogs (in total 81 samples) were transferred to storage at − 80 °C until DNA isolation was performed. The consistency of the faeces was not recorded during the 10-day sampling period, but no dog owner was reporting diarrhoea in their dogs.

After the 10-day sampling period in the home environment, the dogs were subjected to 14–17 h of fasting and then taken to the veterinary clinic at the Swedish University of Agricultural Sciences, Uppsala, Sweden, where they underwent a clinical health examination and had fasting blood samples taken for analysis of serum biochemical and haematological parameters. The study was approved by the Ethics Committee for Animal Experiments, Uppsala, Sweden (C180/12). This prospective study followed the guidelines for reporting observational studies in epidemiology [30] and is an example of an observational study of lean and spontaneously overweight healthy pet dogs that have not undergone any type of intervention, neither weight gain, weight loss or dietary changes. Written consent of the owner was obtained for all dogs.

### Faeces sample preparation

#### DNA isolation

Total DNA was isolated from 0.2 g of faeces using the QIAamp DNA Mini Kit (QIAGEN, GmbH, Hilden, Germany), according to the manufacturer’s protocol, but with a modification for lysis of bacterial cells. Instead of enzymatic lysis of bacterial cell walls, we used bead beating with 0.1 mm zirconium/silica beads (Biospec Products INC, Bartlesville, OK, USA) for 2 × 45 s at setting 5.0 in a FastPrep®-24 benchtop homogeniser (MP Biomedicals, Solon, OH, USA) as bead beating improves the lysis of bacterial cell walls [31]. The isolated DNA was stored at -20 °C until further analysis.

#### Generation of 16S ribosomal RNA gene amplicon libraries

To explore the microbiota composition, 16S rRNA gene amplicons were generated and sequenced by Illumina sequencing [32]. Barcoded polymerase chain reaction (PCR) amplicons were generated with universal primers (515F and 806R, amplifying the V4 region of the 16S gene). PCR reactions were carried out using Phusion® High-Fidelity PCR chemistry (New England Biolabs, Ipswich, MA, USA). After confirmation of positive PCR products, samples were purified with Qiagen Gel extraction kit (Qiagen). The purified products were quantified and samples were pooled into equimolar amounts. The amplicon library was processed with NEBNext Ultra DNA Library prep Kit and the library was then sequenced on a Illumina HiSeq platform 2500 at Novogene (Beijing, China).

The raw sequence reads generated were demultiplexed and assigned to different samples according to the respective barcode. The paired-end sequence reads were merged using FLASH (Version 1.2.7) [33]. Quality filtering of the merged reads was performed according to the Split_Libraries procedure in QIIME (Version 1.7.0) [34]. The quality-filtered sequences were aligned to the Gold database (Release 20110519). Chimera sequences were detected and removed using the UCHIME algorithm (Version 7.0.1001) [35]. UPARSE software (Version 7.0.1001) [36] was used to cluster the remaining sequences into operational taxonomic units (OTUs), using ≥ 97% homology as the threshold for classification as an OTU. For each OTU, a representative sequence was selected for annotation of taxonomic information using the SSU rRNA database SILVA (http://www.arb-silva.se/). Three samples did not pass the quality control prior to sequence analysis and were thus not included in the sequence analysis. Thus the final dataset comprised 78 observations.

### Statistical analyses

Gut microbiota composition, based on OTU data, in the lean and overweight groups of dogs was compared using three multivariate statistical models: principal coordinate analysis (PCoA) based on Bray Curtis distances, analysis of similarity (one-way ANOSIM) with Bray Curtis distance matrices and indicator species analysis (IndVal) to test for multivariate differences between the groups. Similarity index, based on OTU data and Bray Curtis distance matrices for three pairwise comparisons between sampling points (days 1, 5 and 10), was used to express individual variation in each dog over the 10-day period. Mean similarity index for each dog was then used for comparison of temporal variations between the lean and overweight groups of dogs. All multivariate statistical analyses were performed using the statistical software Past, version 4.07 [37].

The alpha diversity of the gut microbiota was assessed with Shannon’s diversity index (reflecting both richness and evenness) and Chao-1 index (reflecting richness only). The diversity indices were generated from OTU data at day 1, 5 and 10 and data were evaluated by mixed-model repeated measures analysis in SAS version 9.4 (SAS Institute Inc., Cary, NC, USA) [38–40] for comparisons of the lean and overweight groups. The mean value (pooled for days 1, 5 and 10) was also compared for lean and overweight groups. The F/B ratio was evaluated by the same procedure. In the mixed-model repeated measures analysis, body condition group (lean and overweight) was defined as an independent variable, and the model analysed the difference between the lean and overweight groups of dogs during the 10-day period (days 1, 5 and 10). The model was thus capable of overall and pair-wise comparisons and corrected for multiple comparisons within the model by Tukey–Kramer adjustment. Logarithmic transformation of raw data was performed to correct for non-normality when needed, based on the appearance of residuals.

Phyla, families and genera detected in over 50% of the observations and with a mean relative abundance of ≥ 1% in the dataset were evaluated by mixed-model repeated measures analysis in SAS as previously described, as were the five highest indicator species in lean and overweight groups, respectively, according to IndVal analysis on OTU data. Genera in the gut microbiota previously shown to differ between lean and overweight dogs in other studies (*Megamonas* and *Roseburia*) [20, 41] were also analysed.

For evaluation of mean diversity (Shannon and Chao-1), mean F/B ratio, similarity index, age and body weight between the lean and overweight groups of dogs, the statistical software Prism (GraphPad Prism 5.0 San Diego, CA), was used. T-tests and Mann–Whitney tests were used for normally and non-normally distributed data, respectively. Level of significance for all statistical analyses (multivariate and univariate) was set to P < 0.05 and results are presented as mean ± SD.

## Results

### Gut microbial diversity in lean and overweight dogs

The sequences analysis generated in average 29,964 (range 12,474–42,484) quality filtered reads per sample. There were no differences in gut microbiota alpha diversity between the lean and overweight groups of dogs with regard to evenness or richness in mixed-model repeated measures analysis or in pooled mean values (Table 1). Individual dogs, irrespective of body condition, displayed variation in alpha diversity, as indicated by a relatively wide range in mean Chao-1 index (122–245) and Shannon index (2.6–3.6).

**Table 1:** Gut microbial diversity (Shannon and Chao-1 index) and similarity index (mean ± SD) in lean and overweight groups of healthy Labrador retriever dogs

OTU data	Lean dogs	Overweight dogs	T-test P-value
	BCS 4–5, n = 12	BCS 6–8, n = 15	
Shannon	3.1 ± 0.3	3.1 ± 0.2	0.59
Chao-1	170 ± 38	177 ± 42	0.78
Similarity index (%)	67 ± 10	67 ± 12	0.40

BCS: Body condition score, OTU: Operational taxonomic unit

### Multivariate comparisons of gut microbiota composition and temporal variations in lean and overweight dogs

**Fig. 1:**
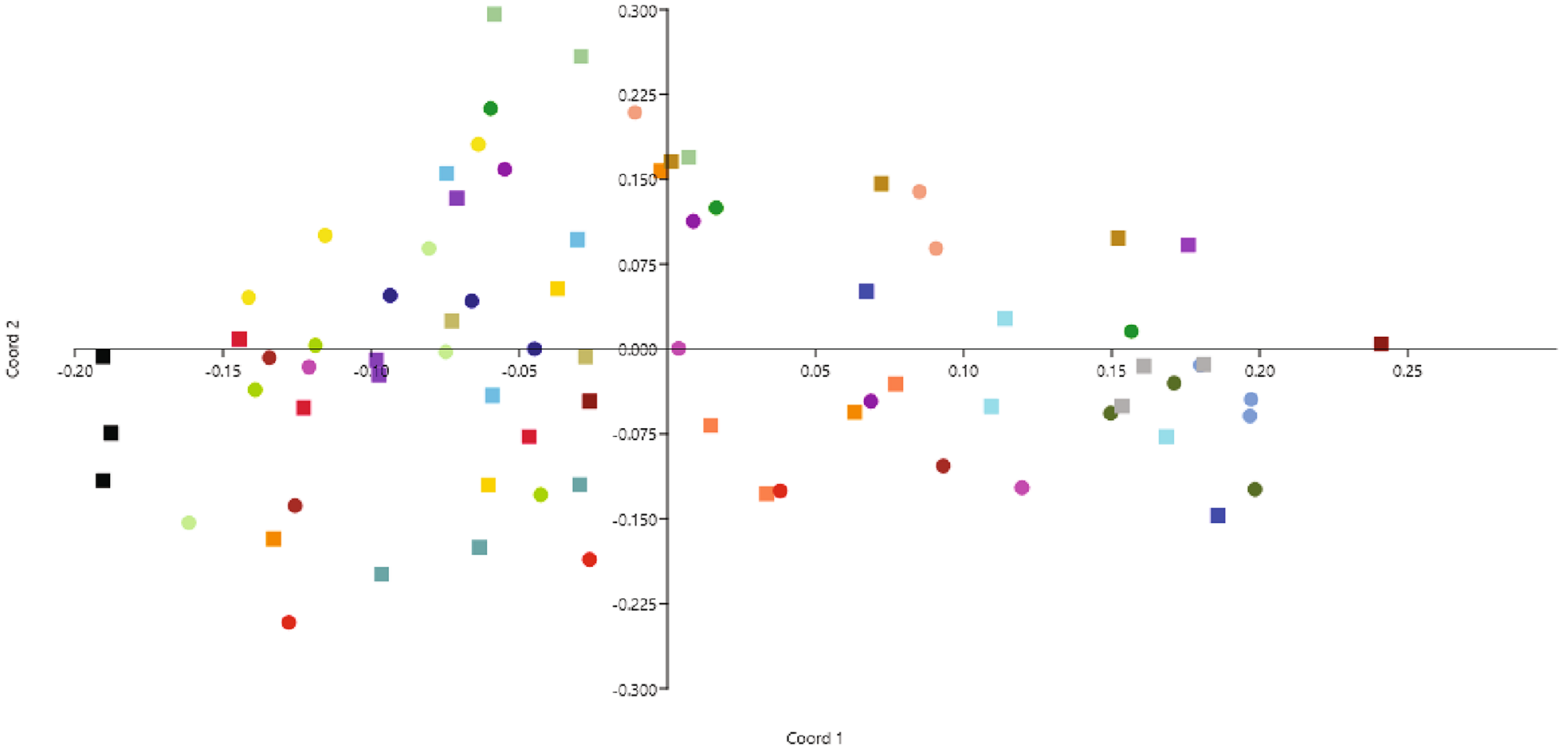
Principal coordinate analysis (PCoA) plot showing multivariate comparisons between lean and overweight groups of dogs. Operational taxonomic units (OTU) for gut microbiota in lean and overweight groups of dogs, sampled during a 10-day period, were subjected to PCoA (1st and 2nd coordinate, x- and y-axis) based on Bray Curtis distances. Lean dogs (BCS 4–5, n = 12) are represented by filled dots and overweight dogs (BCS 6–8, n = 15) by filled squares. Each colour represents one individual dog (three samples per dog; days 1, 5 and 10)

Lean and overweight groups of dogs could not be visually separated by a multivariate model (PCoA). One-way ANOSIM analysis verified that there was no significant multivariate difference in gut microbiota composition between the two body condition groups (P = 0.99) (Fig. 1). Samples from individual dogs (days 1, 5 and 10) mostly clustered in the PCoA plot, and visual inspection of the plot indicated that intra-individual variation in gut microbiota composition over the 10-day period was smaller than inter-individual variation for all dogs. Indicator species analysis of OTU data did not differ between the lean and overweight groups of dogs in mixed-model repeated measures analyses during the 10-day period (P > 0.05 for all). Evaluations based on similarity index showed that the temporal variation in the cohort as a whole was 67 ± 11% (mean ± SD) and that the similarity index did not differ between lean and overweight groups of dogs (Table 1). However, some individual dogs showed larger temporal variations than others, e.g. two lean dogs and four overweight dogs had a similarity index < 60%. Independent of body condition status, different dogs displayed a relatively wide range of the similarity index (range of means 44–85%).

### Univariate comparisons of phylum, family and genus in lean and overweight dogs

**Fig. 2:**
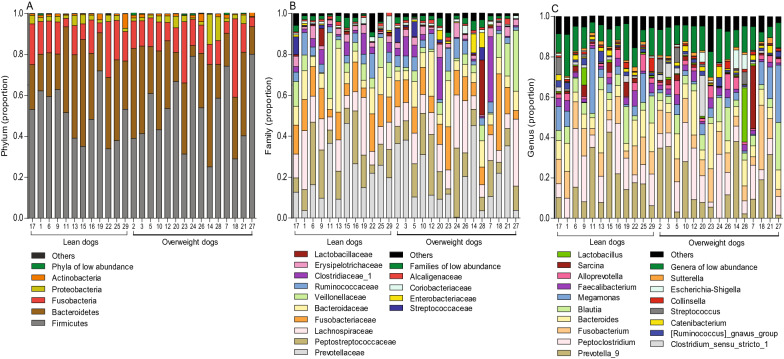
Distribution graphs of gut microbiota composition at phylum, family and genus level in lean and overweight dogs. Gut microbial taxa of phylum (**A**), family (**B**) and genus level (**C**) present in over 50% of the observations are shown in relative abundance (proportions) with the three sampling time points (days 1, 5 and 10) pooled to mean relative abundance for each dog. Microbial phyla, families and genera with low relative abundance (mean < 1%) are grouped as “phyla, families or genera of low abundance” and non-identified taxa are grouped as “others”. Dogs were divided into two body condition groups; lean (BCS 4–5, n = 12) and overweight (BCS 6–8, n = 15). Individual dog ID numbers are shown on the x-axis and dogs are listed according to increasing body condition score (BCS 4–8, left to right). All phyla, families and genera, including those of low abundance, are listed in the raw data in Additional file 2

Phylum, families and genera present in more than 50% of the observations in lean and overweight groups of dogs are shown with pooled relative abundance in distribution graphs in Fig. 2. The microbiota in all dogs was dominated by Firmicutes, Bacteroidetes and Fusobacteria, but with large inter-individual variations in relative abundance of these microbial taxa between dogs (Fig. 2A). The dominant genera for all dogs were *Prevotella*_9 (16%), *Peptoclostridium* (13%), *Fusobacterium* (11%), *Bacteroides* (10%), *Blautia* (7%) and *Megamonas* (6%) but with large inter-individual variations between dogs (Fig. 2C). Phyla, families and genera with a relative abundance ≥ 1% (including *Roseburia*) were analysed in mixed-model repeated measures analyses (except those present in low abundance and others), but specific microbial taxa did not differ between the lean and overweight groups of dogs during the 10-day period (P > 0.05 for all). The proportion of Firmicutes in contrast to Bacteriodetes in individual dogs was evaluated as the F/B ratio (Fig. 2A). A F/B ratio over 3 was slightly more frequent among prominently overweight dogs (BCS > 6), as 50% of those dogs had F/B ratio greater than 3, compared with 24% of lean to slightly overweight dogs (BCS 4–6). The ratio was not significantly different between the lean and overweight groups of dogs in mixed-model repeated measures analysis (P = 0.34). The pooled F/B ratio was 3.1 ± 3.7 in overweight dogs and 2.1 ± 1.2 in lean dogs (P = 0.83).

## Discussion

This study evaluated gut microbiota composition and temporal variations in faeces samples from lean and spontaneously overweight healthy Labrador retriever dogs taken on three occasions over a 10-day period. Similarity index was derived from pair-wise comparisons between the three faeces sampling time points for each dog, which allowed the similarity index to act as an indicator of short-term stability over the 10-day period. Individual variation in short-term stability was relatively high (44–85%) in the dog cohort. However, mean similarity index of gut microbiota did not differ between the lean and overweight groups of dogs. In a previous study investigating the effect of high and low protein diets on gut microbiota composition in lean and obese Beagle dogs, lower similarity index was found in the obese group [26], indicating less resilient gut microbiota when obese dogs were switched between diets. Our findings, on the other hand, suggest that the short-term stability of microbiota was comparable in lean and spontaneously overweight dogs in the absence of interventions.

In the cohort of privately-owned healthy Labrador retriever dogs examined in the present study, lean and overweight dogs did not differ in multivariate comparison of OTU data or in gut microbiota diversity, but individual dogs showed variations in diversity irrespective of body condition status. Previous studies on lean and overweight dogs have also reported no or low separation in microbiota composition according to ANOSIM analyses [20–22], together with no difference in gut microbiota diversity in obese compared with lean dogs [21, 22]. However, other studies in dogs have shown that overweight could have an association with a lower gut microbial diversity. For example, in 24 Beagle dogs, a lower Shannon diversity index was found in overweight compared with underweight dogs [42]. In another study, a high-fat diet together with a moderately increased body condition lead to decreased gut microbiota β-diversity in 24 healthy Beagle dogs after 8 weeks of feeding [24]. Moreover, after a weight-loss intervention in 20 obese pet dogs, the microbial alpha diversity increased when dogs had reached their target weight and the microbiota composition at obese compared with lean state could be separated by ANOSIM analysis [23]. Although the findings from these canine studies are seemingly contradictory, they may suggest that an obese state is needed or that a combination of diet and weight intervention is required to have an impact on gut microbiota composition. It should be emphasised that the present study cohort included only one obese dog (BCS 8), no underweight dog (BCS < 4) and that the observational study design of spontaneous overweight included no type of intervention. Future studies could apply metagenomic or metabolomic techniques to investigate microbial genes or microbial end-products that may differ even when microbiota compositional analyses fail to detect a significant difference between lean and overweight dogs. Moreover, a direct quantitative approach, for example with qPCR, could have revealed potential differences in absolute numbers of bacteria between the groups.

In the present study, the overweight group of dogs had numerically, but not significantly, higher mean F/B ratio than the lean group. In obese humans, gut microbiota composition has been shown to shift to increased relative abundance of Firmicutes and decreased relative abundance of Bacteroidetes, increasing the F/B ratio [18], but conflicting results have also been found [43]. In dogs, it is currently unclear to what extent higher F/B ratio is associated with overweight. It is currently poorly known how proportions of different macronutrients or fibre in dog feed affect the F/B ratio in the canine gut microbiota although studies indicate that proportions of fibre or fat might have an effect [23, 24, 44]. About one third of the included dogs in both lean and overweight groups had fat as their main source of total ME whereas two and four dogs from the lean and overweight group respectively, ate a high-fibre diet (Additional file 1). As diet in home environment was not controlled for in this observational study, it is possible that dietary differences might have influenced the gut microbiota composition irrespective of the overweight state. This, and the absence of body weight interventions, could perhaps partly explain why other studies [23, 24] reported greater differences in F/B ratio between lean and obese states (up to ninefold change) than was observed in the current cohort (1.5 fold change). A cut-off value for F/B ratio in lean and overweight dogs has not yet been proposed, so further research is required to investigate the importance of altered F/B ratio and if needed, set the threshold for high values in dogs.

When planning the present study, there were insufficient data available from previous canine studies to perform power calculations, and all dogs that met the inclusion and exclusion criteria and had dog owners willing to take part in the study were thus enrolled, during a one-year recruiting and sampling period. A post-hoc power calculation on the pooled F/B ratios obtained (mean ± SD) for the lean and overweight groups of dogs (α = 0.05, power = 0.8) showed that a minimum of 23 dogs would have been needed in each group in order to identify statistically significant differences in F/B ratio between lean and overweight dogs. This shows that the current cohort of in total 27 dogs might have been too small in sample size to detect group differences, which is a study limitation. Lack of power or small size effects seem to constitute a general problem in studies investigating gut microbiota alterations in overweight dogs. Strengths of the present study were that repeated faeces sampling and evaluation were performed, all samples were treated according to a controlled study protocol and analysed in one batch.

In tests on the study cohort, none of the tested phylum and family taxa or dominant genera differed in relative abundance between lean and overweight dogs during the 10-day sampling period. It has been shown in other studies that, despite no changes at phylum or family level after a weight-loss intervention in obese dogs, variations in specific bacterial genera may be present [22, 41]. For example, the genus *Roseburia* was found to be more abundant in 18 obese pet dogs and *Megamonas* correlated negatively with weight loss rate in obese dogs during an intervention study with a high-protein high-fibre diet [41]. However, neither *Megamonas* or *Roseburia* were significantly different between lean and overweight groups in the current cohort. Another intervention study investigating 20 obese pet dogs found an increase in *Fusobacterium* spp. and a decrease in *Escherichia coli* at the time point when dogs reached ideal weight [23]. In weight reduction interventions, the included dogs act as their own control which differs from the current observational study design that compares lean and spontaneously overweight dogs. Results generated from these two different study designs should therefore be compared with caution.

The type of feeding in the home environment was not standardized in this study but the dietary history of the dog cohort was quite well known. The frequency at which dogs were fed table scraps, treats and dog chews, for example, did not differ between the lean and overweight groups of dogs (Additional file 1). Gut microbiota composition in dogs can however be influenced by many other different factors than diet, which may also have an impact in this type of study. Arthritis, a common joint disease in obese dogs [45], has been shown to alter the gut microbiota composition in dogs, as has neutering [46, 47]. Our cohort of Labrador retriever dogs was controlled for factors such as breed, sex, age, neutering and health status. Moreover, only healthy dogs without any ongoing veterinary treatments were included. Thus, the results reported in this study were not impacted by any medications, which is otherwise a possible confounder in studies of canine overweight. All dogs were from only one breed and sex, all were intact and free from lameness, which presumably reduced individual variations in gut microbiota composition to some extent, while the repeated faeces sampling approach probably increased the chances of finding potential differences between lean and overweight dogs. Despite the controlled study design, the inter-individual variation in microbiota compositions was quite considerable, and based on visual inspection, inter-individual variation was greater than intra-individual variation in the cohort (Fig. 1).

Cross-sectional studies such as this evaluating gut microbiota composition in spontaneously overweight dogs do not enable conclusions to be drawn regarding causality between excess adiposity and gut microbiota alterations. Longitudinal studies, on the other hand, often use dogs undergoing weight reduction or weight gain, which in dogs involves diet manipulation. It is possible that diet and body condition have a combined effect in changing the gut microbiota in dogs [23, 24, 26, 48] and studies of causality are therefore complicated. Our study is based on a common dog breed, that may often be slightly overweight [49]. Furthermore, the data are generated from dogs living in a regular home environment, not exposed to any dietary intervention or body weight manipulation during sample collection. The results could thus serve as an important basis for future studies of privately owned pet dogs.

### Conclusions

Healthy lean and spontaneously overweight Labrador retriever dogs had comparable gut microbiota composition and short-term stability over a 10-day sampling period. There were no alterations in microbial diversity or in relative abundance of specific taxa at phylum, family or genus level in overweight compared to lean dogs. Our findings suggest that there are few detectable differences in gut microbiota composition between healthy spontaneously overweight and lean dogs by the current method. Future studies including a larger number of dogs, a wider range in BCS and a longer study period might give further information on gut microbiota short-term stability, diversity and F/B ratio in dogs. Moreover, application of metagenomic or metabolomic techniques could be used to investigate microbial genes or microbial end-products that may differ even when microbiota compositional analyses fail to detect a significant difference between lean and overweight dogs.

## Supplementary Information

**Additional file 1. **Background diet in home environment of the 27 Labrador retriever dogs included in the study.

**Additional file 2. **Raw data of all dogs: BCS, OTU, IndVal, Similarity index, Diversity, Phyla, Families and Genera.

## Data Availability

All data generated or analysed during this study are included in this published article and its supplementary information files.
