# Supplementary material for: Composition and short-term stability of gut microbiota in lean and spontaneously overweight healthy Labrador retriever dogs
Source: Acta Vet Scand. 2022 Mar 28;64:8. doi: 10.1186/s13028-022-00628-z (PMC8962211; doi:10.1186/s13028-022-00628-z)
Supplement: Supplementary file 1 — Additional file 1. Background diet in home environment of the 27 Labrador retriever dogs included in the study. [file 13028_2022_628_MOESM1_ESM.docx]

**Additional file 1.** Background diet in home environment of the 27 Labrador retriever dogs included in the study^a^

|  | **Lean dogs** | **Overweight dogs** |
| --- | --- | --- |
|  | BCS (4-5) | BCS (6-8) |
|  | n=12 | n=15 |
| Frequency of scraps, treats and chews^b^ | 6 (4.5-7) | 6 (3.5-7) |
| *pooled scores; median (interquartile range)* |  |  |
| Daily energy intake from commercial diet^c^ | 2:10 | 2:13 |
| *n_75%_:n_100%_* |  |  |
| Wet or dry commercial diet^d^ | 1:11 | 0:15 |
| *n*_wet_:*n*_dry_ |  |  |
| Main protein source in commercial diet^e^ | 8:1:1:1:1:0 | 9:4:1:0:0:1 |
| *n*_C_:*n*_BP_:*n*_B_:*n*_CT_:*n*_S_:*n*_WD_ |  |  |
| Calorie restricted commercial diet^f^ | 2:10 | 4:11 |
| *n*_yes_:*n*_no_ |  |  |
| Main macronutrient source of total ME^g^ | 3:9 | 4:11 |
| *n*_F_:*n*_NFE_ |  |  |

^a^Summary of the background diet received by dogs in their home environment during the 10-day study period when faeces samples were collected. Body condition score (BCS) was clinically evaluated by the same veterinarian (JS) and participating dogs were grouped as lean (BCS 4-5) and overweight (BCS 6-8). In all analyses *P*<0.05 was considered significant. NS; non-significant.

^b^The frequencies with which dogs were given table scraps and rewarded with training treats and dog chews during the 10-day study period were evaluated from daily food diaries provided by the dog owners in the same period. Scraps, treats and chews were scored separately as follows: 0 (never), 1 (once per 10 days), 2 (1-3 times per 10 days), 3 (daily). Scores for scraps, treats and chews were then pooled for each dog and the medians and interquartile ranges for lean and overweight groups were calculated. The difference in total scores between body condition groups was analysed by The Mann-Whitney U test (NS).

^c^The proportion (%) of total daily energy intake coming from a complete commercial diet in home environment was estimated by the dog owner. Group differences analysed by Fisher´s exact test (NS).

^d^Number of dogs fed wet or dry complete commercial diet in the home environment. All dry diets were heat treated while the wet diet received by one dog was a frozen raw formula. Group differences analysed by Fisher´s exact test (NS).

^e^Number of dogs fed a main protein source (in complete commercial diet) from: C=Chicken, BP=Beef and Pork, B=Beef, CT=Chicken and Turkey, S=Salmon, WD=Wisent and Deer*.* Group differences analysed by Chi-square test for trend (NS).

^f^Number of dogs fed a calorie-restricted complete commercial diet in home environment (indicated as a *Light diet* or *weight loosing diet* according to the manufacturer). Group differences analysed by Fisher´s exact test (NS).

^g^Metabolisable energy (ME) mainly from: F=Fat (about 40% of total ME), NFE=Nitrogen free extract (about 40-65% of total ME). No dog had proteins as the main macronutrient source of total ME. Group differences analysed by Fisher´s exact test (NS).
